# What is the status of nucleic acid contamination in 2019-nCOV vaccination sites? Can it be avoided?

**DOI:** 10.1017/S0950268821001394

**Published:** 2021-06-29

**Authors:** Zhanjie Li, Baiyan Zhang, Xinhua Wu, Min Yang, Qi Zhang, Guohua Xiang, Yongxin Chen, Lin Zeng, Dilirena Tayier, Weihong Zhang, Ninghong Song

**Affiliations:** 1 Department of Infection Control, The First Affiliated Hospital of Nanjing Medical University, Nanjing210029, Jiangsu, China; 2 Department of Public Health and Hospital Infection Control, The Affiliated Kezhou People's Hospital of Nanjing Medical University, Kezhou, Xinjiang845350, China; 3 Department of Inspection, The Affiliated Kezhou People's Hospital of Nanjing Medical University, Kezhou, Xinjiang845350, China; 4 Office of the Dean, Jiangsu Shengze Hospital affiliated to Nanjing Medical University, Suzhou215228, Jiangsu, China; 5 Department of Urology, The First Affiliated Hospital of Nanjing Medical University, Nanjing210029, Jiangsu, China; 6 Office of the Dean, The Affiliated Kezhou People's Hospital of Nanjing Medical University, Kezhou, Xinjiang845350, China

**Keywords:** 2019-nCOV, pollution, vaccination

## Abstract

This study aimed to investigate the environmental contamination of nucleic acid at 2019 novel coronavirus (2019-nCOV) vaccination site and to evaluate the effect of improvement to the vaccination process. Nucleic acid samples were collected from the surface of the objects in 2019-nCOV vaccination point A (used between 15 November 2020 and 25 December 2020) and point B (used after 27 December 2020) in a comprehensive tertiary hospital. Samples were collected from point A before improvement to the vaccination process, and from point B (B^1^ and B^2^) after improvement to the vaccination process. The real-time fluorescence polymerase chain reaction method was used for detection. The positive rate of vaccination room was 47.06% (24/51) at point A. No positive result was found in point B^1^ both at working hours (0/27) and after terminal disinfection (0/27). In point B^2^, the positive results were found in vaccine's outer packaging and staff gloves at working hours, with a positive rate of 7.41% (2/27). The positive rate was 0 (0/27) after terminal disinfection in point B^2^. The nucleic acid contamination in the vaccination room of 2019-nCOV vaccine nucleic acid sampling point is serious, which can be avoided through the improvement and intervention (such as personal protection, vaccination operation and disinfection methods).

## Introduction

Severe acute respiratory syndrome coronavirus-2 (SARS-CoV-2) was first reported in January 2020 [1]. The virus is highly and rapidly spread in the population, leading to a prevalence of 2019 novel coronavirus (2019-nCOV) worldwide [2], which has caused great economic loss and life impact [3]. Although China has achieved a phased victory in the prevention and control of the 2019-nCOV epidemic, the international epidemic situation remains extremely severe, and we are still facing a great pressure of ‘preventing external input and internal rebound’ [4]. Although control measures (such as wearing masks, keeping social distance, testing the related people and tracking and isolating the contacts) help to limit the spread of 2019-nCOV and have been implemented in varying degrees, it is not enough to prevent the spread of 2019-nCOV completely [5, 6]. Therefore, 2019-nCOV vaccine is urgently needed to reduce 2019-nCOV-related incidence and mortality rate [5, 7]. Vaccination is a powerful measure to solve or control the further spread and outbreak of 2019-nCOV [8–10]. It has been reported that medical staff with neutralising antibody have a much lower risk of re-infection of SARS-CoV-2 in a short time than those with negative serum [11]. At present, a variety of 2019-nCOV vaccines with proven good effect and safety have been successfully developed at home and abroad [7, 12, 13]. Many countries have begun to carry out 2019-nCOV vaccination [14, 15]. Nevertheless, problems (such as the nucleic acid contamination in the vaccination site of 2019-nCOV vaccine, the link of contamination and how to avoid) have not attracted enough attention. In this study, the environmental contamination in 2019-nCOV vaccination sites were evaluated, the operation links during vaccination that may lead to contamination were analysed, and after improvement to the vaccination process, the effect was assessed, with the purpose to solve this problem.

## Methods

### Objects

**Fig. 1.**
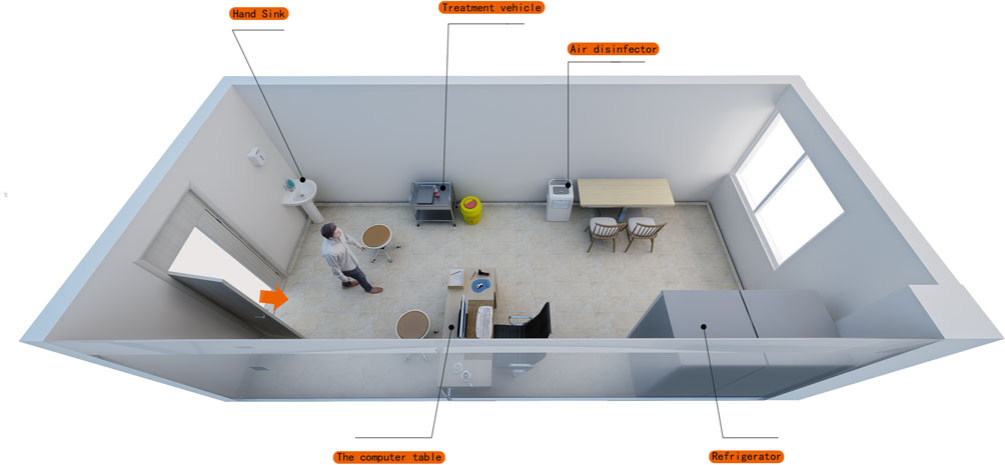
Aerial view of vaccination site (point A).

**Fig. 2.**
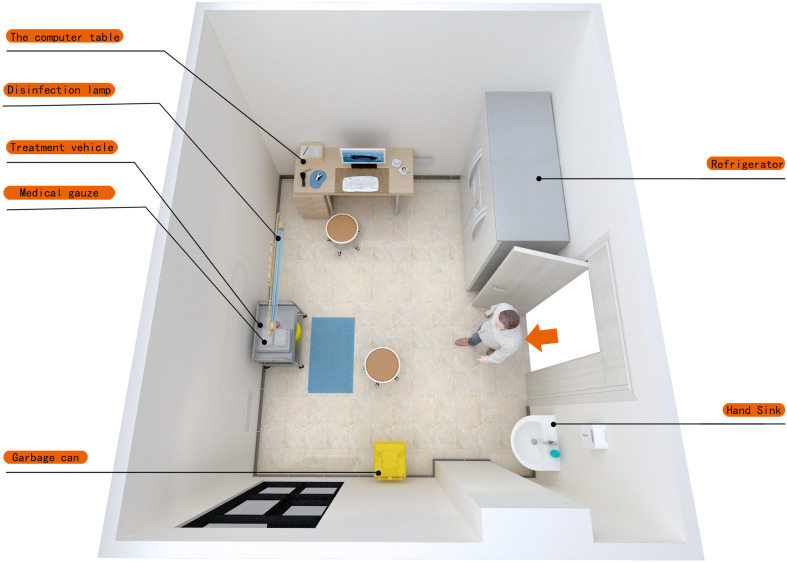
Aerial view of vaccination site (point B^1^).

**Fig. 3.**
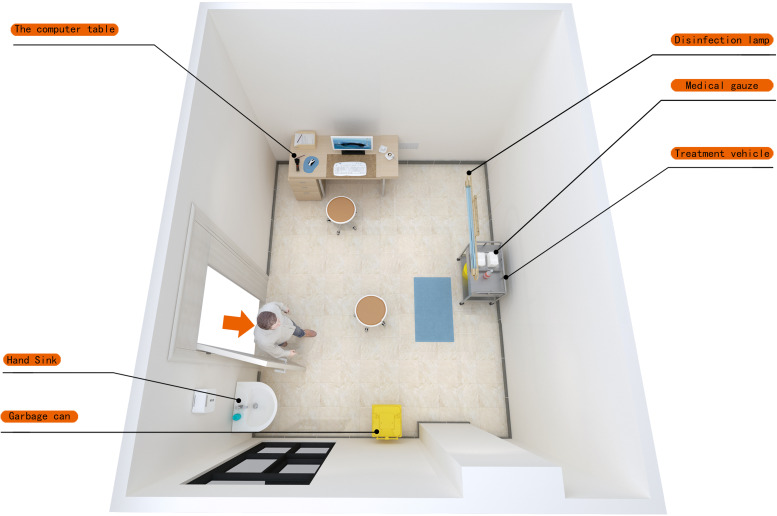
Aerial view of vaccination site (point B^2^).

The environmental substances of 2019-nCOV vaccination site in a comprehensive tertiary hospital were selected as the research object. The vaccination sites were divided into the waiting area, health inquiry area/registration area/informed area, vaccination area, observation area and disposal area for suspected adverse reaction to vaccination. The vaccination-related staff have participated in professional training, and taken up their posts after passing the examination. On 15 November 2020, 2019-NCOV vaccination point A was established in this hospital (aerial view in Fig. 1), which was then moved to point B (aerial view in Figs 2 and 3) on 26 December 2020 (points A and B are two separate areas of different hospital districts). In addition to the movement of the medical refrigerator (for storing vaccines) from point A to point B (for vaccination needs; two vaccination rooms were set in site B), no other item was moved. The vaccination point B was opened on 27 December 2020. A total of 1732 2019-nCOV vaccines were vaccinated in point A from 15 November to 25 December 2020, and 820 2019-nCOV vaccines were vaccinated in point B from 27 December to 02 January 2021. The 2019-nCOV vaccine was the ‘2019-nCOV inactivated vaccine (Vero cell)’ produced by Beijing Institute of Biological Products Co., Ltd., Beijing, China).

### Methods

On 25 December 2020, before the movement of point A, 2019-nCOV nucleic acid on the surface of the subjects in point A was sampled (after terminal disinfection with 500 mg/l chlorine-containing disinfectant). The environmental contamination was analysed, and the possible operation links during 2019-nCOV vaccination that caused environmental contamination were analysed through the sampling results. The possible links during vaccination process were improved in point B. Before the opening of point B on 26 December 2020, and one week after the opening of point B on 2 January 2020, 2019-nCOV nucleic acid on the environmental surface of the vaccination area was sampled (at work hours and after terminal disinfection at the end of work, respectively) to evaluate the effect of improvement to the vaccination process on environmental contamination. The sampling contents include room floor, treatment wheel, desktop, refrigerator, chair, hand sanitiser button, air disinfector, air conditioner's air outlet, code scanning gun, door handle, cuff of vaccination staff, etc.

#### Sampling method

According to the method of monitoring disinfection effect on object surface in WS/T367-2012 ‘technical specification for disinfection of medical institutions’, the disposable virus sampling swab (Kangliyou Medical Development Co., Ltd., Wuhan, China; No.: 20200623) was immersed in the sample preservation solution, which was then directly applied on the surface of the object at the vaccination point, with the sampling swab rotated. Afterward, the sampling swab was broken and placed into the test tube containing 3 ml sample preservation solution (Health Gene Tech Co., Ltd., Ningbo, China; No.: 20200702) for inspection.

#### Detection method

Reverse transcriptase-polymerase chain reaction (RT-PCR) was used for 2019-nCOV nucleic acid detection. The nucleic acid extraction kit and 2019-nCOV nucleic acid detection kit were obtained from Sansure Biotech Co., Ltd. (Hunan, China). The ABI7500 PCR system (Thermo Fisher Scientific, San Jose, CA, USA) was used for product amplification. Primers and probes for the open reading frame (ORFlab) and nucleoprotein (N) regions of 2019-nCOV were selected, with a minimum detection limit of 200 copies/ml.

#### Quality control

There were negative and positive controls (provided by the test kit) in each batch of nucleic acid amplification detection. No CT value or CT >40 was found in all negative control detection channels, and all positive control detection channels were CT ⩽35. The above requirements should be met at the same time in the same experiment; otherwise, this experiment is invalid and needs to be carried out again.

#### Result judgment

The result would be judged as positive if 1ab gene and N gene had both CT ⩽40, with a typical amplification curve. If lab gene CT ⩽40 or N gene CT ⩽40, and there was a typical amplification curve, the sample would be detected again using different reagents, and the result would be judged as positive if the results were the same. If lab gene and N gene both had CT >40 or no CT value, or there was no typical amplification curve, the result would be judged as negative. Lower CT value are indicative of higher nucleic acid concentration of 2019-nCOV.

### Improvement measures

**Fig. 4.**
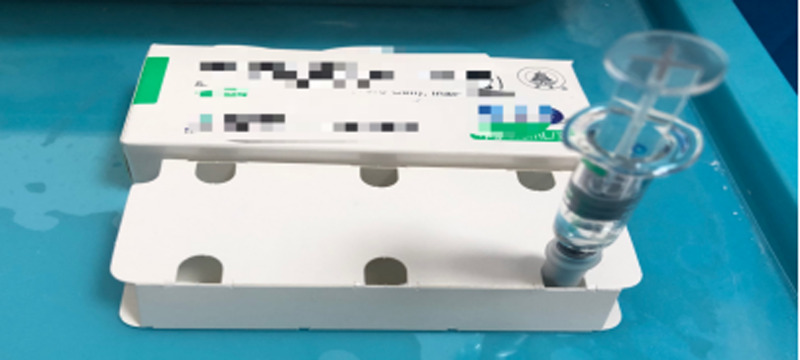
Schematic diagram of needle back to hat with one hand after vaccination.

(1) Personal protections of vaccination personnel were guaranteed (such as medical surgical mask, work clothes, isolation clothes, work cap, gloves (one person for one change) and shoe cover); (2) the treatment tray on the treatment wheel in the vaccination room was completely covered by medical gauze or wet towel soaked with 500 mg/l chlorine-containing disinfectant (or 75% medical alcohol); (3) 2019-nCOV vaccine-contained syringe should be gently vented; even if a small amount of liquid dripped occasionally, it dripped on the treatment tray covered by the wet towel; (4) the needle hat was put upside down in the hole of the box for storing the vaccine; after vaccine injection, the needle was returned to the hat with one hand (Fig. 4) and then put into the sharp instrument box; (5) the towel sheet was laid under the patient's vaccination seat (one shift, one change; the ventilation in the vaccination room was strengthened (ventilation at least three times a day, each time ⩾30 min); (6) the personal protective equipment of the staff should be replaced when they leave the vaccination room; (7) the final disinfection was performed using 500 mg/l chlorine-contained disinfectant and ultraviolet irradiation for 1 h (after the end of each shift).

### Statistical methods

Data were analysed using WPS2019, and the counting data were expressed by cases or percentage.

## Results

### 2019-nCOV nucleic acid-positive rate on the surface of the environment in each area of point A

A total of 51 samples were collected in point A, and the positive rate was 47.06% (24/51). The positive rate of vaccination room was 72.73% (24/33), and the positive rate of sampling in other areas was 0 (Table 1).

**Table 1.** 2019-nCOV nucleic acid-positive rate on the surface of the environment in each area of point A

Sampling area	Number of samples	Positive number	Positive rate
Vaccination room	33	24	72.73%
Health inquiry area	1	0	0
Emergency room	1	0	0
Registration area	4	0	0
Waiting area	5	0	0
Observation area	5	0	0
Public area	2	0	0
Total	51	24	47.06%

### 2019-nCOV nucleic acid-positive sites on the surface of the environment in point A

The 2019-nCOV nucleic acid-positive sites on point A were mainly in the vaccination room. Among these sites, the CT values of 2019-nCOV vaccine droplet, syringe needle and syringe inner wall were all less than 30 in double gene-positive sites. The other double gene-positive sites included the treatment tray, floor, door handle inside the room, refrigerator door handle, leg of seat for patient vaccination and treatment wheel and pen (for staff). The single gene-positive sites included door handle outside the room, door floor, sleeve of vaccination staff, computer keyboard, computer desktop, switch button of mainframe computer, air inlet and outlet of air disinfector, outer packaging of vaccine, hand sanitiser button, outlet of central air conditioner, disinfectant bottle etc. (Table 2).

**Table 2.** 2019-nCOV nucleic acid-positive sites on the surface of environment in point A

Sampling area	Number	Sampling sites	Detection results		Result judgment
			Lab gene	N gene	
Vaccination room	1	2019-nCOV vaccine（Residual droplets in syringe）	19.35	19.04	√(Double)
	2	Syringe needle	25.4	24.35	√(Double)
	3	Syringe inner wall	20.65	19.71	√(Double)
	4	Treatment tray side	34.2	31.48	√(Double)
	5	Treatment tray bottom	35.82	35.73	√(Double)
	6	Floor	38.75	36.5	√(Double)
	7	Door handle (inside)	37.5	35.39	√(Double)
	8	Refrigerator door handle	38.74	35.92	√(Double)
	9	Leg of seat (patient vaccination)	39.91	36.05	√(Double)
	10	Treatment wheel (lower)	35.16	32.69	√(Double)
	11	Pen (for staff)	38.75	35.66	√(Double)
	12	Door handle (outside)	–	39.74	√(Single)
	13	Door floor	–	39.67	√(Single)
	14	Sleeve of vaccination staff	41.67	36.98	√(Single)
	15	Computer keyboard	–	39.5	√(Single)
	16	Computer desktop		38.2	√(Single)
	17	Switch button of mainframe computer	42.76	38.07	√(Single)
	18	Air inlet of air disinfector		37.08	√(Single)
	19	Outlet of air disinfector	42.23	37.42	√(Single)
	20	Vaccine's outer packaging	–	39.95	√(Single)
	21	Hand sanitiser button	–	39.44	√(Single)
	22	Inlet of central air conditioner	38.12	–	√(Single)
	23	Disinfectant bottle (chlorine-containing disinfectant)	–	39.3	√(Single)
	24	Back side of mainframe computer (close to patient seat)		37.34	√(Single)

Note: ‘–’ means that CT value is not detected; ‘ × ’ means that the result is judged as negative; ‘√ (Double)’ means that the result is judged as positive and CT value of both Lab gene and N gene is less than 40; ‘√ (single)’ means that the result is judged as positive and CT value of one of Lab gene or N gene is less than 40.

### 2019-nCOV nucleic acid sampling results before the start of point B

A total of 27 samples were collected. Among these, 22 samples were taken from the vaccination room (refrigerator handle, keyboard, mouse, upper and lower floors of treatment wheel, floor, internal and external door handle, switch, door floor of vaccination room, treatment tray etc.), two samples were taken from the waiting area, two samples were taken from the emergency room and one sample was taken from personal protective equipment (PPE) unloading room. One positive test result was found in the refrigerator handle (CT value of lab gene was 39.95, and no CT value was detected in N gene). In addition to this, all other test results were negative.

### 2019-nCOV nucleic acid sampling results one week after the start-up (improvement to the vaccination process) of point B

A total of 108 samples were collected from the vaccination room one week after the start-up of point B. Briefly, 54 samples (27 samples collected at working hours, and 27 samples collected after terminal disinfection) were collected from point B^1^ and B^2^, respectively. No positive result was found in point B^1^ both at working hours and after terminal disinfection (0/27). In point B^2^, the positive results were found in vaccine's outer packaging (CT value of N gene was 38.09) and staff gloves (CT value of N gene was 38.09) at working hours, with a positive rate of 7.41% (2/27). The positive rate was 0 (0/27) after terminal disinfection in the second vaccination room (Table 3).

**Table 3.** 2019-nCOV nucleic acid sampling results after link improvement in the first vaccination room in point B^1^

Sampling area	Number	Sampling sites	At working hours			After terminal disinfection		
			Detection results		Result judgment	Detection results		Result judgment
			Lab gene	N gene		Lab gene	N gene	
The first vaccination room	1	Floor	–	–	×	–	–	×
	2	Door floor	–	–	×	–	–	×
	3	Door handle (outside)	–	–	×	–	–	×
	4	Door handle (inside)	–	–	×	–	–	×
	5	Patient's seat	–	–	×	–	–	×
	6	Treatment tray (front)	–	–	×	–	–	×
	7	Treatment tray (side)	–	–	×	–	–	×
	8	Treatment tray (bottom)	–	–	×	–	–	×
	9	Treatment wheel (upper)	–	–	×	–	–	×
	10	Treatment wheel (lower)	–	–	×	–	–	×
	11	Code scanning gun	–	–	×	–	–	×
	12	Pen	–	–	×	–	–	×
	13	Hand sanitiser button	–	–	×	–	–	×
	14	Mouse and keyboard	–	–	×	–	–	×
	15	Desktop	–	–	×	–	–	×
	16	Refrigerator handle	–	–	×	–	–	×
	17	Vaccine's outer packaging	–	–	×	–	–	×
	18	Window curtains	–	–	×	–	–	×
	19	Floor towel sheet	–	–	×	–	–	×
	20	Staff shoe cover (bottom)	–	–	×	–	–	×
	21	Sleeve of staff isolation clothing	–	–	×	–	–	×
	22	Staff isolation clothing	–	–	×	–	–	×
	23	Staff gloves	–	–	×	–	–	×
	24	Staff hat	–	–	×	–	–	×
	25	Staff mask	–	–	×	–	–	×
	26	Staff shoe cover (bottom)	–	–	×	–	–	×
	27	Sleeve of staff isolation clothing	–	–	×	–	–	×

Note: ‘−’ means that CT value is not detected; ‘ × ’ means that the result is judged as negative; ‘√ (Double)’ means that the result is judged as positive and CT value of both Lab gene and N gene is less than 40; ‘√ (single)’ means that the result is judged as positive and CT value of one of Lab gene or N gene is less than 40.

## Discussion

RT-PCR and virus gene sequences are gold standards for the diagnosis of 2019-nCOV, and positive results for nucleic acid detection is widely accepted as the diagnosis standard [16, 17]. The inactivated 2019-nCOV vaccine was prepared by culturing and inactivating the wild virus, which has lost its infectivity and pathogenicity. However, its relatively complete viral nucleic acid fragments are still retained. When the vaccination environment is contaminated, the result of nucleic acid detection by PCR (with extremely high detection sensitivity) is likely to be positive if the nucleic acid fragment is sampled before degradation [17]. The virus is not infectious because its nucleic acid in the inactivated vaccine has no activity. Isolation and culture of 2019-nCOV is difficult, and its survival cannot be indicated by 2019-nCOV nucleic acid detection results. Therefore, nucleic acid detection results cannot be used to evaluate the disinfection efficacy [18]. According to ‘Technical Recommendations on Environmental Specimen Monitoring of 2019-nCOV Vaccination Units’ issued by Chinese Center for Disease Control and Prevention on 24 January 2021 [17], routine monitoring of the vaccination environment will interfere with the early warning of epidemic situation monitoring. Hence, it is not recommended for the vaccination units to conduct routine environmental specimen collection and nucleic acid detection. This study was carried out one month before the release of this relevant technical recommendations (starting from 25 December 2020). Although the results of nucleic acid detection at vaccination sites cannot be used to evaluate the disinfection effect, they can be used to assess environmental nucleic acid contamination. Even though it is not recommended to conduct routine environmental nucleic acid detection, it does not mean that we should turn a blind eye to this problem. How to reduce or even avoid environmental contamination at 2019-nCOV vaccination sites is a problem, which calls for attention and needs to be considered (Table 4).

**Table 4.** 2019-nCOV nucleic acid sampling results after link improvement in the second vaccination room in point B^2^

Sampling area	Number	Sampling sites	At working hours			After terminal disinfection		
			Detection results		Result judgment	Detection results		Result judgment
			Lab gene	N gene		Lab gene	N gene	
The second vaccination room	1	Floor	–	–	×	–	–	×
	2	Door floor	–	–	×	–	–	×
	3	Door handle (outside)	–	–	×	–	–	×
	4	Door handle (inside)	–	–	×	–	–	×
	5	Patient's seat	–	–	×	–	–	×
	6	Treatment tray (front)	–	–	×	–	–	×
	7	Treatment tray (side)	–	–	×	–	–	×
	8	Treatment tray (bottom)	–	–	×	–	–	×
	9	Treatment wheel (upper)	–	–	×	–	–	×
	10	Treatment wheel (lower)	–	–	×	–	–	×
	11	Code scanning gun	–	–	×	–	–	×
	12	Pen	–	–	×	–	–	×
	13	Hand sanitiser button	–	–	×	–	–	×
	14	Mouse and keyboard	–	–	×	–	–	×
	15	Desktop	–	–	×	–	–	×
	16	Vaccine's outer packaging	–	38.09	√(Single)	–	–	×
	17	Window curtains	–	–	×	–	–	×
	18	Floor towel sheet	–	–	×	–	–	×
	19	Staff shoe cover (bottom)	–	–	×	–	–	×
	20	Sleeve of staff isolation clothing	–	–	×	–	–	×
	21	Staff isolation clothing	–	–	×	–	–	×
	22	Staff gloves	–	37.91	√(Single)	–	–	×
	23	Staff hat	–	–	×	–	–	×
	24	Staff shoe cover (bottom)	–	–	×	–	–	×
	25	Sleeve of staff isolation clothing	–	–	×	–	–	×

Note: ‘–’ means that CT value is not detected; ‘ × ’ means that the result is judged as negative; ‘√ (Double)’ means that the result is judged as positive and CT value of both Lab gene and N gene is less than 40; ‘√ (single)’ means that the result is judged as positive and CT value of one of Lab gene or N gene is less than 40.

### Environmental contamination at 2019-nCOV vaccination site

This study showed that the nucleic acid-positive rate of environment detection in the 2019-nCOV vaccination room has reached 72.73% (24/33). However, no relevant research on other vaccination sites has been reported at present; therefore, it cannot be directly compared. Growing studies at home and abroad mostly focus on environmental sampling of the isolation ward of 2019-nCOV patients [19–22]. The positive rate of nucleic acid detection from environmental sampling is 2.90% (2/69) to 39.3% (44/112), which can be indirectly compared. It can be clearly seen that the environmental contamination in the 2019-nCOV vaccination room is serious, as evidenced by a much higher positive rate in the 2019-nCOV vaccination room than that in the isolation ward of 2019-nCOV patients. The main contamination sites were treatment wheel (treatment tray), surface of subjects in high contact with hands, air inlet and outlet of air disinfector etc. The detected nucleic acid concentration in some sites (such as vaccine droplets, syringe inner wall and syringe needle) was much higher than that in other sites, which was consistent with the inference that 2019-nCOV vaccine resulted in contamination in other vaccination sites.

### Operation links during vaccination may cause environmental contamination in 2019-nCOV vaccination site

Through the distribution of positive sites, the possible factors of vaccine causing environmental contamination were analysed: (1) the needle with syringe would exude a little during the bumpy transportation process (2019-nCOV vaccine in this study was packed in the syringe with no need to be pumped on site); it is easy to cause staff hand contamination when opening the needle hat if paid no attention; (2) large movement during syringe exhausting and failure to exhaust over the treatment tray may result in contamination in the treatment wheel, ground and staff gloves, sleeves and shoe covers; (3) after the completion of vaccination, a few droplets remained at the tip of the needle; large movement and the action of ‘throwing’ the needle into the sharps box might lead to environmental contamination; (4) cross contamination was caused if the staff in the vaccination room used the contaminated hands (covers) to contact the surface of the computer, the code scanning gun, door handle and other objects; (5) the staff went out of the vaccination room without changing the shoe cover or the bottom of the patient's shoes was contaminated by the ground of the vaccination room, which led to the ground pollution at the entrance of the vaccination room; (6) the use of air disinfector in the vaccination room (at working hours and after terminal disinfection) caused the spread of nucleic acid fragments and expanded the contaminated area (including the inlet and outlet of air disinfector, and air conditioning outlet).

### Analysis of the effects of improvement to the vaccination process

According to the above analysis, improvement to the vaccination process was carried out in the new 2019-nCOV vaccination point B (1.3 Chapter). After the improvement, only the vaccine's outer packaging and gloves were positive at working hours, and all were negative after terminal disinfection. These findings indicated that our improvement to the vaccination process basically avoided the environmental contamination in the 2019-nCOV vaccination point. There are several problems that are easy to be ignored in the whole process of improvement. (1) Personal protection of the vaccination staff: there was no unified international requirement for personal protection of vaccination staff. In this study, positive results were found on the sleeve of the staff's work clothes, indicating that it is necessary for staff to wear isolation clothes, which is in line with the ‘Technical Recommendations on Environmental Specimen Monitoring of 2019-nCOV Vaccination Units’ [17]. In addition, all PPE of staff in the vaccination room should be replaced when leaving the vaccination room. (2) Vaccination operation: the vaccination operation includes exhaustion, medical waste treatment process etc., among these, exhaustion is the ‘culprit’ leading to the contamination of the vaccination site. The vaccination personnel are mainly nurses, who cannot completely extend the liquid preparation habit in clinical treatment to the exhaust operation for 2019-nCOV vaccination. Keeping the action gentle is an important measure to reduce contamination, which has been emphasised in ‘Guidelines for the Prevention of Nucleic Acid Environmental Contamination in 2019-nCOV Vaccination Sites’ issued by Beijing Center for Disease Prevention and Control [23]. (3) Environmental disinfection methods: in addition to surface disinfection using chlorine-containing disinfectant, ventilation, generally speaking, remove virus aerosol quite quickly [24]. A study has shown that in a best-ventilated room, the number of droplets can be reduced by half after 30 s [25]; while in a room with poor ventilation and no ventilation, it may take 1–4 min and 5 min, respectively. Hence, it is necessary to strengthen ventilation. The air disinfector, with access to man−machine co-existence, has been widely used in medical institutions. However, it does not mean that the machine co-exists with any environment. The requirements for circulating air volume of the ultraviolet air disinfector and circulating air disinfector [26, 27] are respectively 10 times and 8 times larger than the applicable volume, which may result in the expansion of environmental contamination. Nevertheless, ultraviolet lamp does not have the above problems, and it can disinfect both the object surface and air, promising to be a choice of terminal disinfection.

## Conclusion

All in all, this study revealed a serious environmental contamination in the vaccination room of the 2019-nCOV nucleic acid sampling points, which should be paid attention. Through the improvement to the vaccination process and intervention (such as personal protection, vaccination operation and disinfection methods), contamination can be avoided, so as to achieve ‘safe vaccination’.
